# Multidimensional Analysis of Physiological Entropy during Self-Paced Marathon Running

**DOI:** 10.3390/sports12090252

**Published:** 2024-09-12

**Authors:** Florent Palacin, Luc Poinsard, Véronique Billat

**Affiliations:** 1 EA 4445—Movement, Balance, Performance, and Health Laboratory, Université de Pau et des Pays de l’Adour, 65000 Tarbes, France; luc.poinsard@gmail.com (L.P.); veroniquelouisebillat@gmail.com (V.B.); 2 Faculty of Sport Science, Université Évry Paris-Saclay, 23 Bd François Mitterrand, 91000 Évry-Courcouronnes, France

**Keywords:** pacing, marathon running, entropy, physiological responses, performance, fatigue, hitting the wall

## Abstract

The pacing of a marathon is arguably the most challenging aspect for runners, particularly in avoiding a sudden decline in speed, or what is colloquially termed a “wall”, occurring at approximately the 30 km mark. To gain further insight into the potential for optimizing self-paced marathon performance through the coding of comprehensive physiological data, this study investigates the complex physiological responses and pacing strategies during a marathon, with a focus on the application of Shannon entropy and principal component analysis (PCA) to quantify the variability and unpredictability of key cardiorespiratory measures. Nine recreational marathon runners were monitored throughout the marathon race, with continuous measurements of oxygen uptake ($\dot{V}$O_2_), carbon dioxide output ($\dot{V}$CO_2_), tidal volume (Vt), heart rate, respiratory frequency (Rf), and running speed. The PCA revealed that the entropy variance of $\dot{V}$O_2_, $\dot{V}$CO_2_, and Vt were captured along the F1 axis, while cadence and heart rate variances were primarily captured along the F2 axis. Notably, when distance and physiological responses were projected simultaneously on the PCA correlation circle, the first 26 km of the race were positioned on the same side of the F1 axis as the metabolic responses, whereas the final kilometers were distributed on the opposite side, indicating a shift in physiological state as fatigue set in. The separation of heart rate and cadence entropy variances from the metabolic parameters suggests that these responses are independent of distance, contrasting with the linear increase in heart rate and decrease in cadence typically observed. Additionally, Agglomerative Hierarchical Clustering further categorized runners’ physiological responses, revealing distinct clusters of entropy profiles. The analysis identified two to four classes of responses, representing different phases of the marathon for individual runners, with some clusters clearly distinguishing the beginning, middle, and end of the race. This variability emphasizes the personalized nature of physiological responses and pacing strategies, reinforcing the need for individualized approaches. These findings offer practical applications for optimizing pacing strategies, suggesting that real-time monitoring of entropy could enhance marathon performance by providing insights into a runner’s physiological state and helping to prevent the onset of hitting the wall.

## 1. Introduction

At the Olympic Games, the marathon continues to hold its mythical status as the ultimate event in the program. This race, long heralded as the pinnacle of human endurance, has evolved from a challenge for elite athletes to a global phenomenon, with over 1.1 million participants annually [1]. Despite its widespread popularity, the marathon remains one of the most grueling tests of physical and mental resilience. A critical aspect of marathon performance is the ability to manage pacing and energy expenditure effectively. Improper pacing can lead to the well-documented phenomenon of hitting the wall, characterized by a dramatic decline in running pace, typically occurring after the 30 km mark. This phenomenon is often attributed to glycogen depletion and associated metabolic shifts, which severely impair a runner’s ability to maintain pace [2,3]. 

Understanding the factors that influence pacing strategies has been a central focus in sports science, particularly in endurance events like the marathon. Effective pacing—the deliberate regulation of speed and effort throughout the race—is crucial for optimizing performance and avoiding premature fatigue [4,5]. Traditionally, marathon runners have been advised to maintain an even pace throughout the race, aiming to distribute their energy expenditure evenly over the entire distance to minimize the risk of early fatigue [5,6,7,8]. However, more recent research suggests that elite marathon runners tend to adopt a more dynamic pacing strategy, characterized by a relatively fast start followed by periods of controlled energy release and variable pacing [9,10]. 

For instance, Billat et al. (2019) found that runners optimize their performance by covering more than 50% of the race distance below their average speed [9]. This strategy involves an initial fast start, which is then followed by a phase of controlled energy management, allowing for variable pacing below the race’s average speed. 

This approach highlights the importance of starting strong while also conserving energy for the later stages of the race, where maintaining pace becomes increasingly challenging. This pacing strategy contrasts with the traditional even-paced strategy and underscores the necessity of adaptability and real-time physiological feedback during the marathon to delay fatigue and avoid hitting the wall [2,5,7,11].

Moreover, Pycke et al. (2022) provide further insight into the effectiveness of a non-uniform pacing strategy, showing that the best marathon performances are achieved not by maintaining a constant pace but by oscillating between extreme values [10]. Their study suggests that these oscillations between faster and slower segments optimize the interplay between aerobic and anaerobic metabolisms, thereby enhancing overall endurance and performance. This strategy, however, requires precise physiological control and the ability to interpret and act on feedback from the body during the race. Abbiss and Laursen (2008) emphasize that pacing is not merely about maintaining a steady pace but involves continuous adjustments based on the athlete’s internal and external environment, underscoring the need for a nuanced understanding of pacing as a dynamic process [11]. 

Despite significant progress in understanding marathon pacing, the interaction between these strategies and the full spectrum of physiological responses during a marathon remains underexplored. Traditionally, studies have focused on isolated variables such as heart rate (HR) and oxygen uptake ($\dot{V}$O_2_) to assess performance. However, recent advancements in wearable technology and data analytics have revolutionized our ability to monitor multiple physiological variables continuously throughout the race. This technological progress presents a unique opportunity to move beyond single-variable analysis and delve into the multidimensional data generated during a marathon, offering deeper insights into performance dynamics.

Leveraging these advancements, our study employs Shannon entropy [12,13]—a concept rooted in information theory—to quantify the variability and unpredictability in physiological signals such as heart rate and oxygen uptake. While Shannon entropy has been widely used in disciplines like neuroscience [14,15] and information theory [12,16], its application in sports science, particularly in endurance sports like marathons, is relatively novel. Most prior research has concentrated on team sports, such as football, where entropy has been utilized to analyze the complexity and unpredictability of team dynamics and player movements [17,18]. In contrast, the use of entropy in individual endurance sports remains underexplored, particularly for understanding how athletes manage physiological stress and pacing over extended periods. By employing entropy analysis in this context, we aim to detect subtle changes in physiological responses that could indicate the onset of fatigue or other critical performance factors during a marathon.

We then apply principal component analysis (PCA) [19,20] to the entropy values of each physiological component of the marathon data. PCA is a powerful tool that reduces the dimensionality of complex datasets, allowing us to identify the most significant patterns of variability in these physiological measures. The objective was to retain as much information as possible by focusing on key sources of variation. If the initial two or three dimensions account for most of the data’s variability, a two- or three-dimensional representation can be constructed to make the data easier to understand. PCA is particularly useful when dealing with large, complex datasets like those collected during a marathon [21]. It enables us to identify relationships between variables, narrowing the focus to the most significant ones for further analysis. The new variables generated by PCA are uncorrelated, making them suitable for subsequent analyses like regression. A key feature of PCA is the creation of a correlation circle, a visual tool that shows the relationship between the original variables and the principal components. Variables that are close to each other on the circle are highly correlated, while those positioned far apart or at right angles are uncorrelated or negatively correlated, respectively. 

In our study, the first two principal components (commonly referred to as F1 and F2) were analyzed to assess their ability to capture the variability in the dataset. To refine the analysis and discern potential differences in PCA patterns according to the marathon’s two halves, we also employed Agglomerative Hierarchical Clustering (AHC) [22]. AHC employs a tree-like structure called a dendrogram to progressively group similar data points, facilitating the identification of clusters within the dataset.

Our multivariate analysis [23], which used PCA and AHC, provided valuable in-sights into the clustering of physiological data (e.g., speed, cadence) relative to the distance run. The analysis revealed two distinct axes: one horizontal axis closely associated with RPE and Rf, and a vertical axis linked with speed, HR, and tidal volume (Vt). Notably, RPE was closely correlated with Rf, while speed, cadence, and HR clustered along the orthogonal F2 axis. Such multivariate analysis could be applied to a larger population to test the hypothesis that some runners exhibit an RPE-Rf-sensitive profile, while others display sensitivity to cadence and Vt. When we classified the runners according to the variables at each 5 km interval, we observed that the classification was consistent for both RPE and speed. Additionally, Runners 6, 7, and 8 exhibited a distinct clustering pattern, while no such similarities were found in the dendrograms for other variables.

These patterns could reveal optimal pacing strategies, offering new perspectives on how athletes manage their energy and avoid the detrimental effects of hitting the wall. 

Through analysis of the cardiorespiratory response and speed entropy, this study aims to further explore the physiological information that runners receive during a real marathon race, specifically focusing on collecting the entire cardiorespiratory response. Expanding on previous methodologies, this study investigates whether the physiological information integrated by runners throughout a marathon is reflected in entropy, as suggested by a prior pilot study [23]. We propose that a multivariate analysis of this entropy matrix can enhance our understanding of hitting the wall by identifying distinct clusters of entropy levels according to the distance run. Additionally, this study examines the multidimensional characteristics of entropy in cardiorespiratory responses to determine whether they provide redundant physiological information during the race. By exploring these factors, we aim to offer new insights into the physiological mechanisms underlying marathon performance.

## 2. Materials and Methods

### 2.1. Subjects

The study included nine recreational but experienced male marathon runners, selected based on their similar physiological and endurance profiles. Their average age was 40.1 years (±10.6), with an average weight of 72.7 kg (±6.5) and height of 178.3 cm (±7.5) (Table 1). 

These runners, who had consistently trained for marathons for over five years, engaged in a weekly routine of three to four training sessions, covering distances between 50 to 80 km. The training regimen included one session of high-intensity intervals (6 × 1000 m at 90–100% of their maximal heart rate) and a tempo run (15–25 km) at 90–100% of their marathon pace. To eliminate potential variability due to gender, only male participants were included in the study [24,25]. All participants provided informed consent, and the study protocol received ethical approval from the relevant institutional review board (CPP Sud-Est V, Grenoble, France; reference: 2018-A01496-49). 

### 2.2. Experimental Design: Marathon Race

Participants competed in an official marathon race (Sénart Marathon, Seine-et-Marne, France), which commenced at 9 a.m. on 1 May 2019. Weather conditions ranged from 11 to 15 °C with an average humidity of 60%, and no precipitation was recorded. Blood lactate levels were measured using a Lactate PRO2 LT-1730 device (ArKray, Kyoto, Japan) immediately after the warm-up (15 min at an easy pace) and three minutes post-race.

### 2.3. Data Collection

Throughout the marathon, respiratory gases, including $\dot{V}$O_2_, carbon dioxide output ($\dot{V}$CO_2_), ventilation [$\dot{V}$E], Vt, Rf, and the respiratory exchange ratio (RER) were continuously recorded using a portable breath-by-breath gas analysis system (K5; Cosmed, Rome, Italy). The system was paired with a Garmin GPS watch (Forerunner 630, Olathe, KS, USA), which measured HR, cadence, and running speed, averaged over five-second intervals. Participants wore masks equipped with inspiratory valves designed to minimize resistance during high-intensity exercise, ensuring comfort and accurate data collection. Given that recent findings have demonstrated that marathon performance depends on pacing oscillations [10], the runners were encouraged to self-pace their run without focusing on the cardio-GPS watch, as its display was intentionally concealed. Hydration and nutrition stations were available every 5 km, providing water, fruit, and sugar, with runners allowed to remove their masks briefly to drink or eat. Each runner drank one glass of water and ate fruits at each hydration point.

### 2.4. Statistical Analysis

All data were analyzed using XLSTAT software (version 2023.2.0.1411; Addinsoft, Paris, France). The results were expressed as mean ± standard deviation (SD), with statistical significance set at *p* < 0.05.

#### 2.4.1. Shannon Entropy

Shannon entropy, a concept from information theory [12,16], is used in this study to quantify the unpredictability or variability within physiological signals like heart rate and oxygen uptake during the marathon. In simple terms, entropy measures the level of “randomness” in a signal. The more unpredictable a signal is, the higher its entropy value [26]. 

For example, consider heart rate data: if a runner’s heart rate fluctuates significantly and unpredictably throughout the race, the entropy of that heart rate signal would be high. Conversely, if the heart rate remains steady with little variation, the entropy would be low. We calculated Shannon entropy using the following formula [13]:

(1) $$H\left(X\right)=-\underset{x_{i}}{\sum }p(x_{i})\log\left(x_{i}\right)$$

Here, $H\left(X\right)$ represents the entropy, and $p\left(x_{i}\right)$ is the probability of each observed state i within the physiological data (e.g., different levels of heart rate). We divided the data into quartiles (Q1, Q2, Q3), with each quartile representing a different “state” of the variable, to understand how the information (or randomness) in the signals evolves throughout the marathon.

In the context of our research, analyzing Shannon entropy allows us to understand how the body’s physiological systems respond to the demands of the marathon, particularly how these responses vary and potentially impact performance.

#### 2.4.2. Multivariate Data Analysis

We examined potential multicollinearity among several key variables, including HR, Rf, running speed, and cadence. Multicollinearity occurs when two or more independent variables in a regression model are highly correlated, which can complicate the interpretation of results. To assess the extent of multicollinearity, we calculated the Variance Inflation Factor (VIF) [27].

The VIF is calculated using the following formula:

(2) $$VIF_{i}=\frac{1}{1-R{²}_{i}}$$

where $R_{2}^{i}$ is the coefficient of determination for regressing the ith against all other independent variables. Essentially, the VIF measures how much the variance of a regression coefficient is inflated due to collinearity with other variables in the model.

VIF values start at 1, indicating no multicollinearity, and have no upper limit. A VIF between 1 and 5 suggests moderate multicollinearity, which is generally acceptable. However, when VIF values exceed 5, this indicates significant multicollinearity, meaning that the coefficients for those variables are less reliable, and the associated *p*-values may be misleading [28]. 

In our analysis, we retained variables with VIF values lower than 5 for further examination through PCA. The rationale was that these variables, despite their high multicollinearity, might capture significant and consistent trends in the physiological responses of all runners. Specifically, variables such as Rf, running speed, Vt, HR, and cadence, which showed consistent patterns in the time series data, were included in the PCA to reduce data dimensionality and identify the most influential components.

This approach allowed us to focus on the key physiological variables that exhibited the strongest and most consistent relationships throughout the marathon, providing a clearer understanding of how these factors contribute to overall performance.

#### 2.4.3. Principal Component Analysis

Principal component analysis [29,30,31] is a powerful statistical technique used to simplify the complexity of multivariate data while preserving as much variability as possible. In this study, PCA was employed to reduce the dimensionality of our dataset, which included various physiological variables measured during the marathon, such as HR, Rf, $\dot{V}$O_2_, running speed, Vt, and cadence. By transforming these variables into a smaller set of uncorrelated components, PCA helps us identify the most significant patterns in the data that contribute to overall performance.

The core idea behind PCA is to project the original data onto a new set of axes, known as principal components. These components are linear combinations of the original variables and are ordered such that the first principal component captures the maximum possible variance in the data, followed by the second principal component, and so on. This process not only reduces the complexity of the data but also highlights the most influential variables in explaining the observed physiological responses during the marathon.

For example, imagine we are tracking multiple physiological variables throughout the race. Each variable provides valuable information, but some may be highly correlated or redundant. PCA combines these related variables into “principal components”, which summarize the essential information while minimizing redundancy. As a result, instead of analyzing each variable independently, we can focus on a few principal components that collectively explain most of the variance in the data.

In our research, after assessing multicollinearity using the VIF, we selected variables with significant multicollinearity for PCA to uncover the underlying structure of the data. The PCA allowed us to identify which physiological responses (such as changes in $\dot{V}$O_2_ or HR) were most significant across different phases of the marathon. These insights are crucial in understanding how various physiological factors interact and influence a runner’s pacing strategy and overall performance.

By reducing the dataset to its most informative components, PCA enabled us to draw clearer, more focused conclusions about the key physiological drivers of marathon performance, paving the way for more targeted strategies to optimize pacing and improve race outcomes.

#### 2.4.4. Agglomerative Hierarchical Clustering

Agglomerative Hierarchical Clustering (AHC) [22,32,33] is a method used to group data points—here, the physiological responses of marathon runners—based on their similarities. Unlike methods that require predefining the number of clusters, AHC starts with each data point as its own cluster and then iteratively merges the closest clusters, forming a hierarchy or “tree” of clusters known as a dendrogram. This approach allows for the exploration of the natural structure of the data without imposing prior assumptions on the number of groups.

AHC operates by calculating the “distance” between clusters, typically using a measure such as Euclidean distance, and merging the pair of clusters that are closest to each other. This process continues until all data points are grouped into a single cluster. The resulting dendrogram provides a visual representation of the clustering process, with the length of the branches indicating the similarity between clusters—the shorter the branch, the more similar the clusters.

In the context of this study, AHC was applied to the physiological data collected from the marathon runners, such as heart rate, respiratory frequency, and oxygen uptake, after reducing the data’s complexity using PCA. By clustering the runners based on these variables, distinct groups of runners with similar physiological responses throughout the race were identified.

This analysis was particularly valuable for revealing how different physiological patterns emerged at various phases of the marathon. For example, AHC grouped runners who displayed consistent heart rate variability and oxygen uptake patterns in the early stages of the race, suggesting a shared pacing strategy, while another cluster included runners whose physiological responses fluctuated more significantly, indicating a different energy management approach.

By applying AHC, the marathon runners were classified into distinct groups based on their entropy profiles and key physiological variables. This clustering provided insights into how different pacing strategies and physiological responses influenced performance outcomes during the race. AHC not only enhanced the understanding of the complex relationships between physiological variables but also offered practical insights for tailoring pacing strategies to individual runners’ physiological profiles.

## 3. Results

Table 2 presents the average speed, HR, and cadence across consecutive 5 km segments for each runner, with particular attention to the point at which each participant hit the wall. In general, a noticeable decrease in speed is observed after the runners reach this critical point. For example, Runner 1’s speed declined steadily from 10.96 km/h in the first 5 km to 8.85 km/h between the 35th and 40th km after hitting the wall at the 27th km. Similar trends were observed in other runners, such as Runner 3, whose speed dropped from 14.63 km/h at 15–20 km to 11.02 km/h at 35–40 km after hitting the wall at the 30th km. Heart rate remained relatively stable for most runners, with minor fluctuations observed across the segments. Cadence also showed slight variations, generally maintaining stability before the wall, followed by a slight decrease in some cases after the wall. Runner 4, for instance, experienced a decrease in cadence from 86.4 ppm at 15–20 km to 77.8 ppm at 35–40 km after hitting the wall at the 26th km.

Table 3 presents the entropy values for running speed, HR, and cadence across consecutive 5 km segments for all runners, with a focus on the changes observed around the point at which each participant hit the wall. Across all runners, a general trend of increased entropy in speed, HR, and cadence is observed as they approach and pass the critical wall point, indicating a rise in variability and instability in their performance metrics.

For most runners, speed entropy tends to increase around the point where they hit the wall. For example, Runner 1’s speed entropy increases from 1.6 ± 0.6 between 10–15 km to 2.31 ± 0.33 between 25–30 km, shortly after hitting the wall at the 27th km. Similar patterns are seen in Runner 4, whose speed entropy rises from 1.48 ± 0.59 between 10–15 km to 2.23 ± 0.5 between 25–30 km, hitting the wall at the 26th km.

Heart rate entropy also shows noticeable changes around the wall. For instance, Runner 9, who hit the wall at the 27th km, experiences an increase in HR entropy from 1.03 ± 1.12 between 0–5 km to 2.44 ± 0.78 between 15–20 km, reflecting greater variability in heart rate as the race progresses. This pattern is consistent across several other runners, including Runner 7, whose HR entropy increases from 1.72 ± 1.03 between 0–5 km to 1.94 ± 0.79 between 10–15 km, before stabilizing slightly.

Cadence entropy generally follows a similar trend, with most runners showing increased variability in their cadence as they hit the wall. Runner 2, for example, shows an increase in cadence entropy from 2.12 ± 0.67 between 0–5 km to 2.79 ± 0.42 between 30–35 km, hitting the wall at the 33rd km. Runner 8 also displays a steady increase in cadence entropy, reaching a peak of 2.95 ± 0.16 between 35–40 km, after hitting the wall at the 34th km.

**Figure 1:**
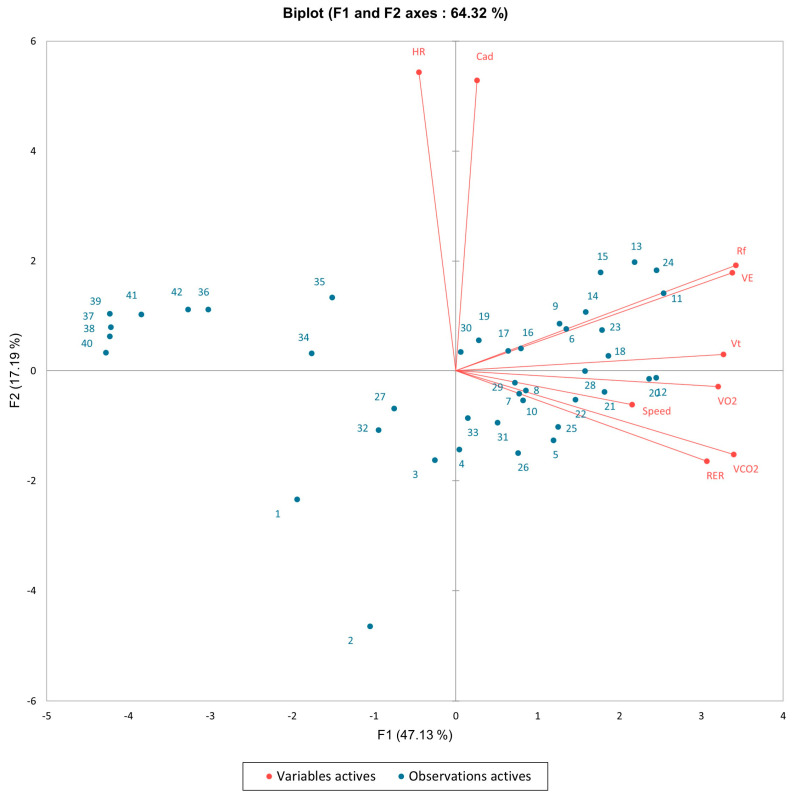
Biplot from the principal component analysis (PCA) illustrating the relationship between physiological and cadence responses during the marathon, with Runner 3 serving as a representative example for all nine marathoners. The red vectors represent the variables, including heart rate (HR), cadence (Cad), oxygen uptake ($\dot{V}$O_2_), carbon dioxide output ($\dot{V}$CO_2_), respiratory frequency (Rf), tidal volume (Vt), respiratory exchange ratio (RER), ventilation ($\dot{V}$E), and speed. The blue points represent the observations corresponding to the different kilometers of the marathon. The F1 axis explains 47.13% of the variance, while the F2 axis accounts for 17.19%, together capturing a total of 64.32% of the variance in the data.

Figure 1 presents the biplot from the PCA applied to the physiological and cadence responses during the marathon, using Runner 3 as a representative example. The analysis shows that the first principal component (F1), which accounts for 47.13% of the total variance, is strongly associated with the entropy variance of $\dot{V}$O_2_, $\dot{V}$CO_2_, Vt, running speed, and RER. These variables are closely aligned along the F1 axis, indicating that they share a significant amount of variance.

The last kilometers of the marathon (36 to 42) are located on the biplot in the opposite direction to the vectors representing the respiratory variables ($\dot{V}$O_2_, $\dot{V}$CO_2_, Vt, and RER) along the F1 axis. 

The second principal component (F2), which explains 17.19% of the variance, is associated with HR and cadence, which are closely aligned with this axis. The entropy of Rf also follows a different pattern, with a closer alignment to the F2 axis in the two-dimensional projection. Cadences are also notably aligned along the F2 axis.

**Figure 2:**
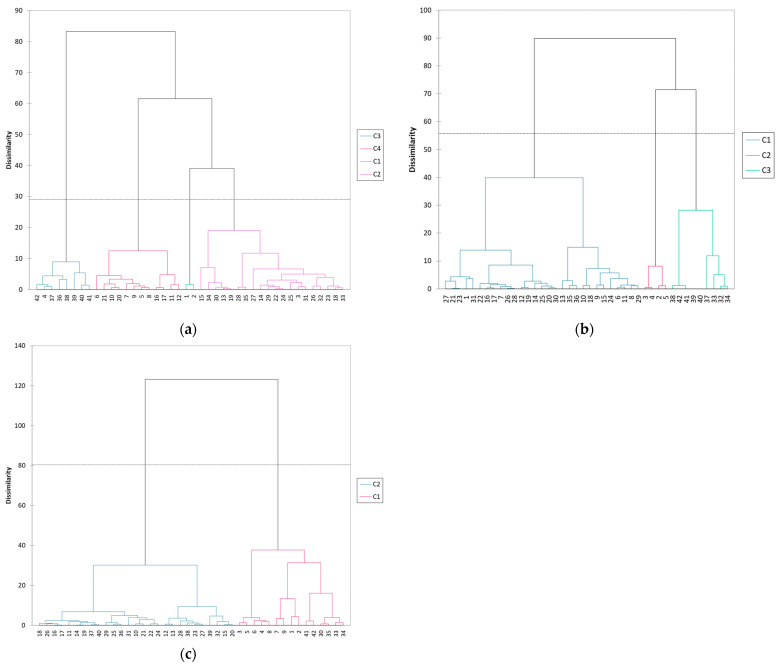
The dendrogram for the classification of entropy profiles during the marathon using Agglomerative Hierarchical Clustering. The y-axis represents the dissimilarity between clusters, while the x-axis shows the kilometers run by each participant. (**a**) The dendrogram for Runner 6, showing four distinct clusters, representative of four other runners with a four-class profile. (**b**) The dendrogram for Runner 2, showing three clusters, typical of three runners with a three-class profile. (**c**) The dendrogram for Runner 8, displaying two clusters, indicating a two-class profile unique to this runner.

Figure 2 illustrates the results of the Agglomerative Hierarchical Clustering (AHC) performed to classify the entropy profiles of marathon runners, based on their physiological responses throughout the race.

For Runner 6 (Figure 2a), the AHC identified four distinct clusters (C1, C2, C3, C4), with the transition from C3 to C4 occurring at a dissimilarity value of approximately 80, indicating a noticeable change in physiological responses. This four-class profile, shared by three other runners, suggests distinct stages or phases throughout the marathon.

For Runner 2 (Figure 2b), the AHC revealed three distinct classes (C1, C2, C3), with a major separation between C2 and C3 at a dissimilarity value of approximately 70. The overall dissimilarity reached up to 100, highlighting three key phases in the runner’s physiological response, representing the initial, middle, and final stages of the marathon. This three-class profile was also observed in two other runners.

For Runner 8 (Figure 2c), the AHC identified two main classes (C1 and C2), with the overall dissimilarity exceeding 120, the highest among the three runners. The transition between these two classes occurred at a dissimilarity value of approximately 50, marking a significant shift in physiological responses, possibly corresponding to the early and late stages of the marathon.

## 4. Discussion

The present study explored the intricate relationship between pacing strategies and physiological responses during a marathon, employing Shannon entropy as a novel tool to quantify the complexity of these responses for each marathoner. The findings of this study provide new insights into how marathon runners manage their physiological resources over the course of the race, and how this management may influence their performance, particularly in relation to the phenomenon of hitting the wall. 

In this study, we applied PCA to explore the multidimensional nature of physiological entropy during a marathon, focusing on key variables such as $\dot{V}$O_2_, $\dot{V}$CO_2_, HR, Rf, cadence, and running speed. PCA is a powerful tool for reducing the dimensionality of complex datasets, allowing us to identify the most significant patterns of variability in these physiological measures. Our analysis revealed that these variables were distributed across two main axes (F1 and F2), each representing distinct dimensions of physiological variability.

Notably, the F1 axis captured the maximal entropy variance for $\dot{V}$O_2_, $\dot{V}$CO_2_, Vt, running speed, and RER. However, the later stages of the marathon were positioned on the opposite side of this axis, indicating a divergence in physiological states as fatigue set in. The alignment of these respiratory variables on the F1 axis underscores their critical role in the physiological stress experienced during the final kilometers of the race, a phenomenon consistent with the onset of hitting the wall as documented by Coyle (2007) [34]. The concentration of these variables on the same axis suggests that they are closely linked in their response to the increasing metabolic demands as glycogen stores deplete and fatigue sets in. 

Interestingly, while $\dot{V}$O_2_ and $\dot{V}$CO_2_ were aligned on the F1 axis, $\dot{V}$CO_2_ does not follow the respiratory frequency, despite typically inducing an increase in Rf [35]. This discrepancy could be due to the low value of RER reported in prior studies, which is often associated with lower blood lactate accumulation during endurance events [36,37]. Although a marathon can elicit $\dot{V}$O_2_ max during the race, this state is only achieved for a small percentage of the marathon duration (4 ± 4% of the run time) [38]. Additionally, it is known that hyperventilation induced by increased $\dot{V}$CO_2_ can lead to a slowing of EEG activity, potentially resulting from cerebral ischemic anoxia due to hypocapnic cerebral vasoconstriction, direct effects of hypocapnia on nerve cells, or cerebral alkalosis [39]. These findings suggest that the respiratory parameters are closely linked in their response to the increasing metabolic demands during the later stages of the marathon. Assessing the probability of changes in O_2_ and CO_2_ states in relation to speed and cadence alterations might provide insights into the central governor’s [40,41] role in integrating physiological signals. This role includes modulating pace based on the relative changes in $\dot{V}$O_2_ and $\dot{V}$CO_2_ states, particularly when these variables approach their maximums, as measured in a preliminary maximal test [42,43].

In contrast, the entropy variance for HR, cadence, and Rf was associated with the F2 axis, indicating that these variables may reflect separate dimensions of physiological response during the marathon. The differentiation of entropy measures across multiple dimensions suggests that various physiological systems respond differently to the demands of marathon running. While respiratory variables showed increased entropy variability towards the end of the race, HR, cadence, and Rf exhibited distinct patterns, particularly along the F2 axis. This suggests that these variables may maintain a more stable response throughout the marathon, potentially reflecting the body’s effort to preserve homeostasis under conditions of prolonged physical stress. These findings are consistent with research by Laursen and Rhodes (2001), which highlighted the relatively stable nature of heart rate during steady-state endurance exercise, contrasting with the more variable responses seen in respiratory parameters as the body attempts to cope with the increasing fatigue [44].

The second major finding of our study is that ACH effectively identified distinct patterns of physiological entropy throughout the marathon, demonstrating notable inter-individual variability in how race segments are physiologically categorized. Among four of the nine participants, AHC identified four distinct classes of entropy, reflecting a more complex physiological response pattern. These classes often exhibited overlapping segments, blending kilometers from the start, middle, and end phases of the marathon. This suggests that these runners experienced more gradual transitions in their physiological responses, without clear-cut shifts between race phases, potentially reflecting varied strategies in energy management or differing responses to fatigue.

Conversely, in two other participants, the AHC revealed two distinct entropy classes, which marked a clear physiological division between the early and later stages of the race. This two-class distinction likely reflects the characteristic shift in physiological demands that occur when runners transition from relative metabolic stability to the more severe metabolic stress associated with hitting the wall, typically around the 30 km mark [45]. These runners appeared to have relatively stable physiological responses during the initial stages of the race, followed by a pronounced shift as they neared this critical point.

For the remaining three runners, the analysis revealed three distinct classes, corresponding to the early, middle, and late stages of the race. This three-phase pattern suggests a more gradual adaptation to the physiological demands of the race, with runners modulating their energy expenditure in the middle phase before the physiological strain intensifies. Studies have suggested that such a pacing strategy, involving a slight reduction in speed during the middle stages of a race, can help conserve glycogen stores and delay the onset of significant fatigue [7,11]. These runners may have employed more controlled pacing strategies, making subtle adjustments as they progressed, which allowed them to distribute the metabolic load more evenly over the course of the marathon.

These findings suggest that the segmentation of the marathon, from an informational entropy perspective, is subject to considerable individual differences. The varying number of classes required to categorize race segments highlights the personalized nature of physiological responses during prolonged endurance exercise. This study underscores the potential utility of individualized entropy analysis of physiological metrics as a tool for optimizing marathon performance. By tailoring race strategies based on the unique classification of entropy across different race segments for each runner, there is an opportunity to enhance pacing, energy management, and overall race efficiency.

The objective of research focused on running performance is to define the optimal speed variation based on both aerobic and anaerobic physiological characteristics. This variation synchronizes two metabolic pathways to achieve the highest average speed in the marathon, aided by recovery possibilities [10]. Human metabolism operates through aerobic and anaerobic systems simultaneously to respond to fluctuations in power demand. If we liken human metabolism to a hybrid engine, anaerobic metabolism functions like an electric battery, enabling acceleration, while aerobic metabolism serves as the thermal engine, recharging the battery. Running too fast risks hitting the “marathon wall” due to factors such as decreased blood glucose, which is essential for muscle contraction even at low speeds.

Muscular fatigue also contributes as the muscles lose contractility and elasticity after the approximately 35,000 steps required to complete a marathon in 3 h and 15 min, with a step frequency of 3 Hz. Additionally, fluctuations in body temperature, as seen in the 2018 Boston Marathon with hypothermia cases, can increase the perception of strain, leading to uncontrollable speed drops. Nybo and Nielsen (2001) used electroencephalography (EEG) and electromyography to measure brain and muscle activity during exercise in elevated temperatures [46]. They concluded that heightened perceived effort was more linked to cerebral activity than muscle activity, finding a linear relationship between brain activity and rising body temperature. A temperature rise to 40–41 °C, recorded at marathon completion, should not occur as early as the 28th kilometer. For reference, an increase of +10 beats per minute in heart rate for the same speed can indicate elevated body temperature.

Digestive discomfort, often caused by excessive fluid intake or overly concentrated sports drinks, can also affect performance [47,48]. High entropy values are linked to changes in velocity, and using velocity entropy as a reference may be more beneficial than relying solely on maximal aerobic speed. Since not all optimal speed variation parameters can be controlled, trusting one’s sensations becomes paramount. Speed regulation involves a constant interplay between a predictive pace plan (feed-forward) and continuous adjustment (biofeedback). One hypothesis suggests that the central governor in the brain oversees this process, treating the body as a complex system, adjusting skeletal muscle recruitment to manage pacing strategies [49]. Fatigue can be understood as a sensory perception arising from the integration of physiological, biochemical, and sensory feedback, which may or may not correlate with changes in muscle force production.

Athletes adopt a range of paces depending on the event’s duration. According to the central governor model, pace adjustments and exercise cessation are part of a dynamic regulatory strategy aimed at protecting the body. This subconscious pacing strategy appears oscillatory, while changes in RPE occur more gradually, reflecting biological demands tied to maintaining homeostasis or approaching exercise termination [40,50,51,52]. The central nervous system (CNS) integrates feed-forward information with afferent sensory feedback from metabolic and other changes in various organs, resulting in these pacing strategies [50]. However, this assertion remains unproven in the context of real marathon running. EEG offers new opportunities to measure brain activity non-invasively and is increasingly used in exercise research [53,54]. Human EEG has been shown to synchronize with muscle contractions [55,56,57] and gait phases [58].

The findings of this study reinforce the understanding that optimal marathon performance requires a delicate balance between aerobic and anaerobic metabolism. The ability to switch between these metabolic pathways is crucial for maintaining speed while avoiding premature fatigue, analogous to a hybrid engine alternating between electric and thermal energy sources. This concept is well-documented in endurance sports research [59,60].

While our study did not directly measure brain activity, prior research shows that EEG can provide insights into how the CNS contributes to fatigue and pacing strategies [61]. The alignment of physiological variables with distinct axes in the PCA suggests that central regulation mechanisms may play a role in pacing adjustments, especially in the later stages of the marathon. This aligns with the central governor model, where the brain modulates effort to keep physiological systems within safe limits [41,59].

Future research could benefit from incorporating EEG measurements alongside entropy analysis to further explore the CNS’s role in endurance performance. This could lead to more effective strategies for managing pace and avoiding fatigue in marathon running [62,63].

These findings offer several practical implications for marathon runners, coaches, and sports scientists. The identification of distinct entropy profiles in cardiorespiratory responses provides a valuable tool for optimizing pacing strategies. By understanding the evolution of a runner’s physiological responses throughout a marathon, tailored strategies can be developed to enhance performance and minimize premature fatigue. Additionally, real-time monitoring of cardiorespiratory entropy could allow runners to dynamically adjust their pace during the race. For example, reducing pace in response to early signs of excessive energy expenditure could conserve energy for the critical later stages, improving overall performance and reducing the risk of exhaustion.

Insights into the changing entropy profiles across the race also offer a nuanced understanding of fatigue management. By pinpointing stages where significant physiological changes are likely, targeted interventions can help maintain endurance and focus during these critical periods. Coaches could leverage entropy data to create personalized training regimens that align with a runner’s specific physiological characteristics, enhancing training effectiveness.

The potential integration of advanced technologies such as EEG and artificial intelligence for real-time physiological data analysis could lead to wearable devices that provide immediate feedback and performance optimization. This technological advancement could democratize sophisticated data analytics, benefiting runners of all levels.

Lastly, understanding variations in entropy profiles could aid in injury prevention. Identifying patterns linked to overexertion or injury risk allows for strategic adjustments in training or race plans, mitigating these risks. However, further research is needed to validate these results on larger, more diverse populations before implementing them on a large scale.

## 5. Conclusions

This study provides valuable insights into the complex relationship between pacing strategies and physiological responses during a marathon, with a particular focus on the role of respiratory variables and the application of Shannon entropy and principal com-ponent analysis (PCA) to quantify these dynamics. The findings suggest that respiratory measures, especially oxygen uptake, carbon dioxide output, and tidal volume, are critical in managing physiological demands; however, during the later stages of the marathon, these measures diverge from earlier trends, as reflected in their positioning on the opposite side of the F1 axis, indicating a shift in the body’s metabolic state as the risk of hitting the wall increases. The distribution of entropy variance across multiple axes for different physiological variables highlights the complexity of the body’s response to prolonged endurance exercise and suggests that multiple physiological factors must be considered to optimize performance and manage fatigue effectively.

Furthermore, the application of Agglomerative Hierarchical Clustering (AHC) provided additional insights into individual variability in physiological responses. The AHC analysis revealed distinct patterns among runners, with some exhibiting two, three, or four distinct entropy classes throughout the marathon. This variability indicates that runners experience physiological transitions at different points during the race, reflecting varied pacing strategies and fatigue responses. The ability of AHC to classify these patterns underscores its potential as a tool for identifying personalized pacing strategies and for understanding how different runners manage their energy expenditure over the course of the marathon.

However, several limitations should be acknowledged. The relatively small sample size and focus on recreational marathon runners may limit the generalizability of these findings to broader populations, particularly elite athletes who may exhibit distinct physiological responses and pacing strategies. Additionally, while the study suggests a potential role for central regulation mechanisms in pacing strategies, this hypothesis was not directly tested. Future research would benefit from integrating neurophysiological assessments, such as EEG, to more comprehensively explore the central nervous system’s role in endurance performance.

## Figures and Tables

**Table 1:** Subjects’ ages, personal records, and the year of their best performances.

Runners id	Age (Years)	Fastest Marathon Times (Years)	Sénart Marathon Times (2019)
1	58	03h27′32″ (2013)	04h30′34″
2	47	02h59′22″ (2016)	03h32′07″
3	29	02h57′03″ (2015)	03h14′13″
4	36	03h27′58″ (2017)	03h51′13″
5	43	02h44′00″ (2015)	03h13′42″
6	23	03h22′40″ (2019)	03h22′40″ *
7	44	03h34′57″ (2017)	03h34′57″ *
8	47	03h12′48″ (2016)	03h31′34″
9	34	02h50′00″ (2019)	02h50′00″ *

* Subjects who achieved their personal best during the Sénart Marathon.

**Table 2:** Summary of average speed (km/h), heart rate (HR: beats per minute), and cadence for each 5 km segment. The cadence values are reported as steps per minute (ppm) for one leg, as recorded by Garmin. The values are expressed as mean ± standard deviation.

id	Variable	Wall (km)	0–5	5–10	10–15	15–20	20–25	25–30	30–35	35–40	40–42
1	Speed	27	10.96 ± 0.34	11.07 ± 0.21	10.77 ± 0.21	10.72 ± 0.34	10.36 ± 0.28	9.83 ± 0.42	9.57 ± 0.39	8.85 ± 0.87	9.71 ± 0.45
	HR		155.30 ± 3.29	159.80 ± 1.60	156.30 ± 1.57	157.70 ± 1.30	160.40 ± 1.85	159.80 ± 1.10	159.80 ± 0.84	151.90 ± 8.43	161.50 ± 2.83
	Cadence		87.80 ± 0.76	88.20 ± 0.45	88.00 ± 0.79	86.60 ± 0.65	86.20 ± 1.44	86.10 ± 0.89	84.30 ± 0.97	82.40 ± 5.86	81.00 ± 2.12
2	Speed	33	13.96 ± 0.28	13.76 ± 0.47	13.47 ± 0.39	13.44 ± 0.36	13.29 ± 0.33	12.87 ± 0.27	12.33 ± 0.30	11.67 ± 0.84	13.41 ± 1.25
	HR		159.50 ± 2.09	163.10 ± 0.55	158.60 ± 1.85	158.50 ± 1.46	159.60 ± 2.04	158.20 ± 1.60	158.00 ± 1.12	158.30 ± 0.84	162.50 ± 2.12
	Cadence		89.00 ± 0.61	88.00 ± 0.71	88.10 ± 1.29	87.90 ± 0.82	88.40 ± 0.82	87.30 ± 1.44	87.50 ± 0.35	87.80 ± 0.45	90.00 ± 1.41
3	Speed	30	13.78 ± 0.71	14.41 ± 0.32	14.63 ± 0.30	14.28 ± 0.28	14.07 ± 0.21	13.23 ± 0.39	12.32 ± 0.80	11.02 ± 0.32	13.55 ± 0.76
	HR		152.10 ± 9.77	161.50 ± 2.29	166.10 ± 2.53	163.30 ± 1.60	162.70 ± 0.91	160.00 ± 2.45	156.00 ± 1.27	151.60 ± 1.29	161.00 ± 0.71
	Cadence		82.90 ± 0.55	84.00 ± 0.50	83.80 ± 0.67	83.40 ± 0.65	84.10 ± 0.65	82.80 ± 0.27	81.70 ± 0.27	80.20 ± 0.91	81.75 ± 1.06
4	Speed	26	11.03 ± 0.28	11.07 ± 0.31	10.77 ± 0.24	10.62 ± 0.15	10.52 ± 0.34	9.78 ± 0.26	9.59 ± 0.31	8.30 ± 1.36	9.96 ± 0.97
	HR		155.49 ± 2.95	159.63 ± 1.41	156.50 ± 1.08	157.97 ± 1.57	160.21 ± 1.46	160.80 ± 0.74	159.90 ± 0.58	151.51 ± 9.80	162.19 ± 1.24
	Cadence		87.92 ± 0.44	88.00 ± 0.42	87.96 ± 0.54	86.42 ± 0.75	86.47 ± 1.09	85.40 ± 0.99	85.03 ± 0.78	77.83 ± 8.76	84.17 ± 0.87
5	Speed	30	16.76 ± 0.46	16.09 ± 0.52	13.80 ± 0.19	13.76 ± 0.43	14.05 ± 0.47	12.90 ± 1.19	13.15 ± 0.50	12.94 ± 0.56	12.53 ± 0.31
	HR		156.60 ± 4.20	160.90 ± 0.89	162.70 ± 1.40	166.30 ± 0.97	168.70 ± 0.97	167.90 ± 1.34	169.60 ± 0.96	172.20 ± 2.75	177.00 ± 1.41
	Cadence		88.10 ± 0.96	86.00 ± 0.35	85.50 ± 0.35	85.50 ± 1.06	84.90 ± 0.42	85.50 ± 0.94	84.90 ± 0.55	84.60 ± 0.65	84.25 ± 0.35
6	Speed	34	12.93 ± 0.19	13.22 ± 0.11	12.98 ± 0.21	12.97 ± 0.21	12.98 ± 0.22	12.58 ± 0.42	12.24 ± 0.55	11.05 ± 0.21	11.58 ± 0.13
	HR		148.90 ± 4.55	157.40 ± 1.14	159.40 ± 0.74	161.60 ± 0.65	163.40 ± 1.39	163.60 ± 1.14	162.90 ± 1.56	158.80 ± 1.35	161.25 ± 2.47
	Cadence		93.00 ± 0.35	92.40 ± 0.42	92.30 ± 0.91	92.50 ± 0.61	92.60 ± 0.96	91.50 ± 0.50	91.70 ± 0.57	92.10 ± 0.22	92.50 ± 0.71
7	Speed	27	12.57 ± 0.23	12.76 ± 0.20	12.17 ± 0.54	12.12 ± 0.43	12.04 ± 0.24	11.57 ± 0.43	11.13 ± 0.45	10.75 ± 0.38	
	HR		157.70 ± 3.35	161.90 ± 2.22	162.50 ± 0.35	163.10 ± 1.52	164.00 ± 3.46	162.10 ± 2.82	155.60 ± 2.82	152.00 ± 5.68	
	Cadence		86.30 ± 0.27	86.30 ± 0.45	85.80 ± 0.57	85.70 ± 0.57	85.70 ± 0.27	85.80 ± 0.27	85.70 ± 0.27	85.83 ± 0.29	
8	Speed	34	13.17 ± 0.72	13.45 ± 0.26	12.84 ± 0.33	12.82 ± 0.31	12.52 ± 0.46	12.03 ± 0.26	11.81 ± 0.28	11.51 ± 0.27	11.40 ± 0.33
	HR		163.00 ± 7.90	167.20 ± 1.04	166.60 ± 0.96	167.40 ± 2.51	170.10 ± 0.22	169.90 ± 1.56	169.00 ± 1.46	169.70 ± 1.57	170.25 ± 2.47
	Cadence		92.60 ± 0.22	92.60 ± 0.42	92.50 ± 0.79	92.20 ± 0.45	91.50 ± 1.06	91.80 ± 0.27	91.90 ± 0.82	92.50 ± 0.79	92.50 ± 0.00
9	Speed	27	15.38 ± 0.35	15.40 ± 0.22	15.10 ± 0.21	14.82 ± 0.30	15.14 ± 0.43	14.64 ± 0.51	14.74 ± 0.14	14.15 ± 0.20	14.86 ± 0.11
	HR		137.30 ± 11.83	147.70 ± 0.57	146.80 ± 3.31	150.00 ± 2.62	146.50 ± 2.24	149.00 ± 1.41	151.30 ± 1.15	150.10 ± 1.95	154.25 ± 1.77
	Cadence		87.00 ± 0.61	86.80 ± 0.27	86.10 ± 0.22	85.30 ± 1.10	85.00 ± 0.35	83.90 ± 0.74	85.00 ± 0.61	84.90 ± 0.55	85.00 ± 0.71

Note: To obtain the total cadence, the values should be multiplied by two (e.g., 89 ppm corresponds to 178 ppm for both legs). Runner 7 accidentally stopped his watch at km 37.

**Table 3:** Summary of entropy values for speed, heart rate (HR), and cadence across consecutive 5 km segments during the marathon. The entropy values represent the complexity and variability of each physiological variable, providing insights into the dynamic fluctuations experienced by each runner throughout the race. The values are expressed as mean ± standard deviation, illustrating the average level of entropy and its variability within each 5 km segment.

id	Variable	Wall (km)	0–5	5–10	10–15	15–20	20–25	25–30	30–35	35–40	40–42
1	Speed	27	1.16 ± 1.24	0.96 ± 0.69	1.6 ± 0.6	2.41 ± 0.46	2.1 ± 0.49	2.31 ± 0.33	1.84 ± 0.56	1.32 ± 0.9	2.01 ± 0.8
	HR		1.23 ± 0.79	1.78 ± 0.57	1.63 ± 0.4	1.84 ± 0.6	1.8 ± 0.64	1.13 ± 0.71	1.72 ± 0.83	1.45 ± 1.06	1.16 ± 1.03
	Cadence		1.51 ± 0.86	0.82 ± 0.69	1.47 ± 0.68	1.53 ± 0.45	2 ± 0.48	2.11 ± 0.67	2.72 ± 0.55	2.38 ± 0.37	2.97 ± 0.48
2	Speed	33	1.49 ± 0.63	1.26 ± 0.75	1.59 ± 0.7	1.91 ± 0.73	1.63 ± 0.71	1.7 ± 0.42	1.29 ± 0.5	0.75 ± 0.74	0.88 ± 1.2
	HR		2.79 ± 0.16	0.69 ± 0.97	1.63 ± 1.03	1.86 ± 0.45	1.57 ± 0.32	1.59 ± 0.74	1.93 ± 0.58	1.87 ± 0.51	1.55 ± 1.34
	Cadence		2.12 ± 0.67	2.57 ± 0.63	2.91 ± 0.39	2.98 ± 0.25	2.38 ± 1.02	2.45 ± 0.58	2.79 ± 0.42	2.56 ± 0.4	2.31 ± 1.07
3	Speed	30	1.48 ± 0.67	1.28 ± 0.55	1.29 ± 0.57	1.3 ± 0.47	1.56 ± 0.36	1.08 ± 0.64	1.13 ± 0.58	0.15 ± 0.25	1.23 ± 1.08
	HR		1.14 ± 0.71	1.91 ± 0.17	1.18 ± 0.78	1.68 ± 0.48	1.71 ± 0.38	1.34 ± 0.72	0.88 ± 0.67	1.02 ± 0.82	1.26 ± 1.09
	Cadence		2.63 ± 0.44	1.89 ± 0.57	1.65 ± 0.75	2.92 ± 0.16	2.08 ± 0.73	1.95 ± 0.75	1.42 ± 0.29	1.36 ± 0.77	1.63 ± 0.59
4	Speed	26	1.69 ± 1	1.33 ± 0.67	1.48 ± 0.59	1.56 ± 0.74	1.89 ± 0.87	2.23 ± 0.5	1.86 ± 0.79	0.87 ± 0.99	0.2 ± 0.34
	HR		1.1 ± 1.02	2.15 ± 0.53	1.3 ± 0.85	1.48 ± 0.86	1.92 ± 0.6	1.79 ± 1.05	1.14 ± 0.68	1.06 ± 0.99	1.12 ± 1
	Cadence		2.05 ± 1.08	0.45 ± 0.43	1.24 ± 0.79	1.03 ± 1.14	2.18 ± 0.48	2.21 ± 0.58	2.56 ± 0.74	2.24 ± 0.85	1.79 ± 1.55
5	Speed	30	0 ± 0	0.74 ± 1.15	1.79 ± 0.36	1.79 ± 0.4	1.58 ± 0.22	1.37 ± 0.79	1.69 ± 0.73	1.4 ± 1.02	0 ± 0
	HR		0.47 ± 0.51	1.54 ± 0.27	0.89 ± 0.85	0.69 ± 0.65	2.05 ± 0.31	2.1 ± 0.41	1.31 ± 0.69	0 ± 0	0 ± 0
	Cadence		0.28 ± 0.31	1.4 ± 0.54	1.61 ± 0.5	2.33 ± 0.48	2.1 ± 0.52	2.23 ± 0.28	2.49 ± 0.24	1.22 ± 0.88	0 ± 0
6	Speed	34	1.87 ± 0.66	1.22 ± 0.35	1.7 ± 0.42	2.09 ± 0.26	2.05 ± 0.64	1.55 ± 0.5	1.1 ± 0.73	0.24 ± 0.37	1.31 ± 1.34
	HR		0 ± 0	1.22 ± 0.76	2.15 ± 0.49	1.3 ± 0.65	0.94 ± 0.69	0.84 ± 0.77	0.9 ± 0.77	1.87 ± 0.3	1.98 ± 0.49
	Cadence		1.56 ± 0.91	2.14 ± 0.29	2.41 ± 0.77	2.57 ± 0.37	2.49 ± 0.42	2.25 ± 0.29	2.43 ± 0.25	2.41 ± 0.31	2.57 ± 0.68
7	Speed	27	2.76 ± 0.39	1.9 ± 0.66	2.63 ± 0.72	2.05 ± 0.62	2.58 ± 0.34	2.47 ± 0.37	2.17 ± 0.58	0.83 ± 0.45	
	HR		1.72 ± 1.03	1.67 ± 0.72	1.94 ± 0.79	1.5 ± 0.39	1.04 ± 1.05	1.38 ± 0.83	1.49 ± 1.13	1.35 ± 1.17	
	Cadence		2.15 ± 0.46	2.16 ± 0.37	2.15 ± 0.31	2.26 ± 0.2	2.32 ± 0.32	2.39 ± 0.32	1.21 ± 0.31	1.08 ± 0.3	
8	Speed	34	1.12 ± 1.14	0.52 ± 0.81	1.48 ± 0.85	1.72 ± 0.92	1.57 ± 0.4	1.63 ± 0.46	1.98 ± 0.52	1.29 ± 0.88	0.84 ± 1.12
	HR		1.06 ± 1.09	2.09 ± 0.7	2.08 ± 0.52	1.73 ± 0.56	1.68 ± 0.31	1.5 ± 0.98	1.57 ± 0.89	1.95 ± 1.12	1.61 ± 1.64
	Cadence		2.17 ± 0.3	2.42 ± 0.28	2.41 ± 0.42	2.84 ± 0.26	2.84 ± 0.13	2.82 ± 0.14	2.92 ± 0.17	2.95 ± 0.16	2.86 ± 0.17
9	Speed	27	1.39 ± 1.33	1.2 ± 0.6	2.02 ± 0.58	1.85 ± 0.36	2.14 ± 0.42	2.1 ± 0.86	1.62 ± 0.58	0.68 ± 0.67	1.42 ± 1.04
	HR		1.03 ± 1.12	1.43 ± 0.77	2.22 ± 0.61	2.44 ± 0.78	2.19 ± 1.2	1.99 ± 1	1.48 ± 0.81	1.95 ± 0.6	0.87 ± 0.98
	Cadence		0.31 ± 0.35	0.14 ± 0.33	1.09 ± 1.12	2.71 ± 0.44	2.91 ± 0.38	2.44 ± 0.75	2.34 ± 0.38	2.7 ± 0.32	2.51 ± 0.27

Note: Runner 7 accidentally stopped his watch at km 37.

## Data Availability

The data presented in this study are available upon request from the corresponding author.
